# Association of ESR and FOXP3 gene polymorphisms with outcome of ovarian stimulation in infertile females undergoing IVF

**DOI:** 10.1186/1755-8166-7-S1-P61

**Published:** 2014-01-21

**Authors:** Arun Kiran Patnam, R Vinu, J Vijayalakshmi, P Venkatachalam, G Usha Rani

**Affiliations:** 1 Department of Human Genetics, Sri Ramachandra University, Porur, Chennai, India; 2 Department of Biomedical Sciences, Sri Ramachandra University, Porur, Chennai, India; 3 Department of Obsteritics and Gynecology, Sri Ramachandra University, Porur, Chennai, India

**Keywords:** *ESR*, *FOXP3*, infertility, *IVF*, poor ovarian response

## Background

Estrogen receptor (ER) gene plays a major role in folliculogenesis, maturation of oocytes and fertilization of embryo. FOXP3 gene is a crucial regulatory factor for the development and function of Treg cells; it takes part as a central role in the induction and maintenance of fetal-maternal immunologic tolerance. Therefore, the present study was aimed to investigate the association of ESR and FOXP3 gene polymorphisms in infertile women who are undergoing in vitro fertilization (IVF) with poor ovarian reserve upon controlled ovarian hyperstimulation (COH) induced by follicle-stimulating hormone (FSH).

## Materials and methods

Genomic DNA was extracted from EDTA-peripheral blood by using high salting-out method, polymerase chain reaction-restriction fragment length polymorphism (PCR-RFLP) analysis was done followed by DNA sequencing to confirm the results for ESR1 gene on intron 1, PvuII T/C (rs2234693) and XbaI A/G (rs9340799); ESR2: RsaI G/A (rs1256049) on exon 5; and FOXP3 gene: HaeIII T/C (rs2294021) single-nucleotide polymorphisms (SNPs). Genotyping was carried out in infertile female (n=25) undergoing in-vitro fertilization (IVF) with poor ovarian reserve and healthy fertile controls (n=25).

## Results

Statistical analysis of ESR 1 (rs2234693) gene polymorphism showed a significant association (p<0.05) between cases and controls. Whereas, ESR 1 (rs9340799), ESR2 (rs1256049) and FOXP3 (rs2294021) genotypes showed no significant association (P>0.05) in cases and controls.

## Conslusion

As polymorphism rs2234693 (PvuII) showed a significant association in dominant model (Odds ratio: 3.19 (95%CI: 1.00-10.17); P: 0.04), genetic variability of the Estrogen receptor gene may exert an indirect effect on the pregnancy outcome of IVF patients by affecting the development of the follicles, oocytes and embryos. Still, further studies are necessary to confirm our findings with larger sample size that might improve our understanding of ESR1 gene polymorphisms and its importance for advancing infertility diagnoses, treatment and genetic counselling.

